# Activation Mechanism of Ammonium Fluoride in Facile Synthesis of Hydrated Silica Derived from Ferronickel Slag-Leaching Residue

**DOI:** 10.3390/molecules29040905

**Published:** 2024-02-18

**Authors:** Xuqin Duan, Yu Zhang, Dong Li, Tong Liu, Yanjun Jiang

**Affiliations:** School of Civil and Resource Engineering, University of Science and Technology Beijing, Beijing 100083, China

**Keywords:** ferronickel slag, leaching residue, hydrated silica, low-temperature roasting, ammonium fluoride

## Abstract

A novel process for the synthesis of hydrated silica derived from ferronickel slag (FNS)-leaching residue was proposed in this study. The products of the purification of hydrated silica with 99.68% grade and 95.11% recovery can be obtained through ammonium fluoride (NH_4_F) roasting, followed by the process of water leaching, ammonia precipitating, and acid cleaning under the optimized conditions. The effects of NH_4_F mass ratio, roasting temperature, and roasting time on the water-leaching efficiency were investigated in detail. The thermodynamic and X-ray diffraction analyses indicated that the amorphous silica in FNS-leaching residue was converted to water-soluble fluoride salts ((NH_4_)_2_SiF_6_) during the roasting process, which are also supported by the scanning electron microscopy and thermogravimetry analyses. The Si–O bonds in amorphous silica could be effectively broken through the ammonium fluoride activation during a low-temperature roasting process. This work provides a meaningful reference for further studies on the facile synthesis of hydrated silica with similar mineral compositions.

## 1. Introduction

Ferronickel slag (FNS) is a kind of industrial solid waste created during the production of nickel alloys and stainless steel [1,2]. Generally, 14 tons of FNS may be generated for producing one-ton ferronickel. In China, more than 30 million tons of FNS are released annually, which has become the fourth largest type of solid waste in the smelting industry after blast furnace slag, steel slag, and red mud [3,4,5]. The chemical components of FNS mainly include SiO_2_, MgO, CaO, Al_2_O_3_, Fe_2_O_3_ and so on, where the amorphous minerals as the major component are greater than 50% content. In addition, crystalline minerals such as enstatite, forsterite, and diopside also exist in ferronickel slag [6,7,8].

FNS can be used in many fields, such as metal recycling, glass, ceramics, refractory materials, etc. The recycling of valuable metals in FNS is preferred due the economic benefits of these resources [9,10,11]. However, limited to the low utilization ratio, large amounts of FNS-leaching residue are dumped or landfilled, occupying a large land area as well as polluting the environment [12,13,14,15]. In consideration of the high potency of amorphous silica contained in FNS acid-leaching residue, the synthesis of hydrated silica might be fit for its potential utilization, which appears to be a possible technology employing frugal resources and that is environment friendly.

The flame pyrolysis method and precipitation method are the well-known processes for the synthesis of spherical colloidal silica nanoparticles [16]. For the fumed silica produced by flame pyrolysis, though its purity and particle distributions are more attractive, the complex and expensive manufacturing process limits its industrial application scope [17,18,19]. In contrast, the precipitated silica is relatively cheaper to produce and can be realized on a larger scale. Hence, the synthesis of hydrated silica by the precipitation method might be more suitable for utilizing the abundant leaching residue of ferronickel slag [20,21]. To guarantee the quality of hydrated silica products, separating amorphous silica from impurity minerals is one of the most critical steps for synthesis. However, the conventional roasting–leaching process is limited to its high roasting temperature and energy consumption. Furthermore, the conventional process cannot effectively destroy the silicon-oxygen tetrahedron structure of amorphous silica and remove the impurity minerals in FNS-leaching residue. The quality and purity of hydrated silica products can hardly be guaranteed in a conventional process [22,23]. In recent years, much attention has been paid to the use of ammonium fluorine salts such as NH_4_F to realize low temperature roasting [24,25,26]. The activation roasting process is based on the difference in ammonium fluorine reaction difficulty between amorphous silica and impurity minerals. Water-soluble products and high reactivity would be obtained through the low temperature roasting, and then the silica components can be extracted by water leaching [27,28]. In view of this, ammonium fluorine salts might lower the roasting temperature and improve the roasting performance of FNS-leaching residue, which have positive impacts on synthesizing hydrated silica. Nevertheless, there are only a few reports about correlational studies, and especially, the activation mechanism of ammonium fluoride in the roasting process of FNS-leaching residue is still unclear.

In this study, a new environmental and economic process using NH_4_F as activators to realize low-temperature roasting was proposed for the synthesis of hydrated silica. The effects of ammonium fluoride roasting conditions on the leaching efficiency of amorphous silica were studied in detail, and the NH_4_F activation mechanism for low-temperature roasting was also systematically analyzed.

## 2. Results and Discussion

### 2.1. Characterization of Ferronickel Slag-Leaching Residue

The chemical multi-element analysis results of FNS-leaching residue are shown in Table 1. It can be seen that the content of SiO_2_ is 81.34%, which is the major composition of FNS-leaching residue. The impurity contents such as metallic oxide (CaO, Fe_2_O_3_, Al_2_O_3_, MgO, etc.) in FNS-leaching residue are relatively lower.

**Figure 1:**
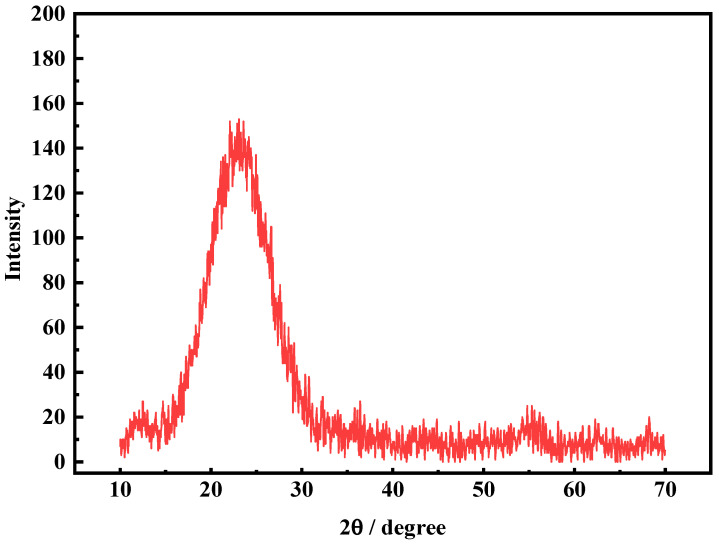
XRD analysis of the FNS-leaching residue.

The XRD patterns of the FNS-leaching residue are presented in Figure 1. It can be seen that the XRD curves demonstrate broad diffraction peaks at the 2θ range of 20~30°, which are the typical characteristic peaks of the glass phase, indicating that amorphous silica is the abundant existence form of silicon dioxide [29]. The lack of metallic phase such as calcium, aluminum, iron, etc., might be attributed to the low content and amorphous silica coating. The results of XRD and chemical multi-element analysis indicate that the amorphous silica is the abundant composition of FNS-leaching residue.

**Figure 2:**
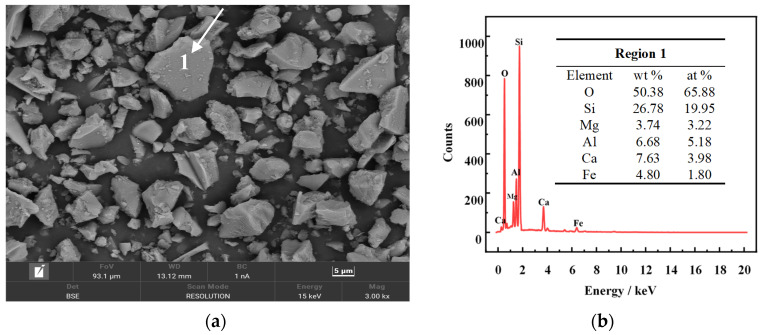
SEM images (**a**) and EDS spectrum (**b**) of the FNS-leaching residue.

As shown in Figure 2a, the SEM images of FNS-leaching residue demonstrate that the particles with irregular shape and sharp edge are equipped with a dense structure, which is detrimental to the dissolving and leaching process. The EDS spectrum of region 1 in SEM images shows that silicon and oxygen are the major elements, which is inconsistent with the results of the aforementioned analyses. Furthermore, the existence of magnesium, aluminum, calcium, iron, etc., elements might be ascribed to the undissolved minerals during the sulphuric acid leaching, which are also the impurity elements needing to be removed for the synthesis of hydrated silica.

**Figure 3:**
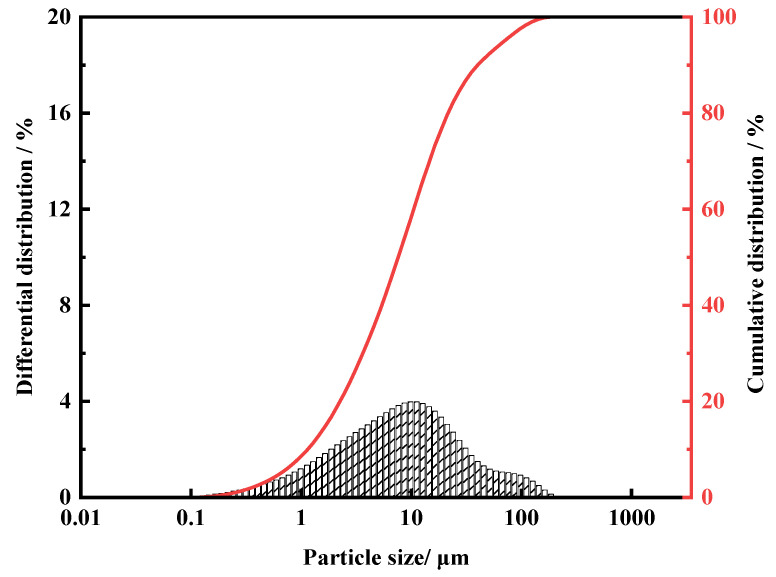
Volume-weighted particle size distributions of FNS-leaching residue samples.

The particle size distribution curve of FNS-leaching residue is presented in Figure 3. The particle size range is relatively narrow with a fine size, where the D_50_ and D_90_ are 8.67 μm and 45.8 μm, respectively. 

### 2.2. Facile Synthesis of Hydrated Silica through Ammonium Fluoride Roasting

For the entire synthesis process of hydrated silica, ammonium fluoride roasting is the most critical step. Therefore, the effects of the ammonium fluoride roasting conditions on the leaching efficiency of silica were first studied in detail. 

**Figure 4:**
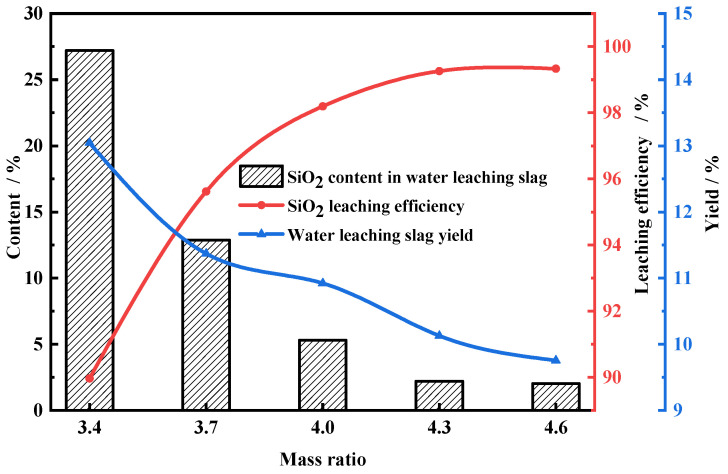
Leaching efficiency of FNS-leaching residue for different mass ratios of NH_4_F. Roasting conditions: temperature, 140 °C; roasting time, 2 h. Water-leaching conditions: leaching time, 1.5 h; liquid–solid ratio, 10: 1; stirring speed, 300 r/min.

The relationship between the NH_4_F mass ratio and the water-leaching efficiency of FNS-leaching residue is shown in Figure 4. The mass ratio of NH_4_F is referred to the ratio between the mass of NH_4_F and FNS-leaching residue. The SiO_2_-leaching efficiency increased gradually from 89.96% to 99.26% when the NH_4_F mass ratio increased from 3.4:1 to 4.3:1; meanwhile, the yield and SiO_2_ content of water-leaching slag correspondingly decreased. The results indicate that the amount of ammonium fluoride obviously influences the leaching efficiency. With the mass ratio increasing from 4.3:1 to 4.6:1, the water-leaching efficiency remained constant. Hence, the mass ratio of NH_4_F fixed at 4.3:1 is the optimal dosage and enough for the fluoride reaction of amorphous silica.

**Figure 5:**
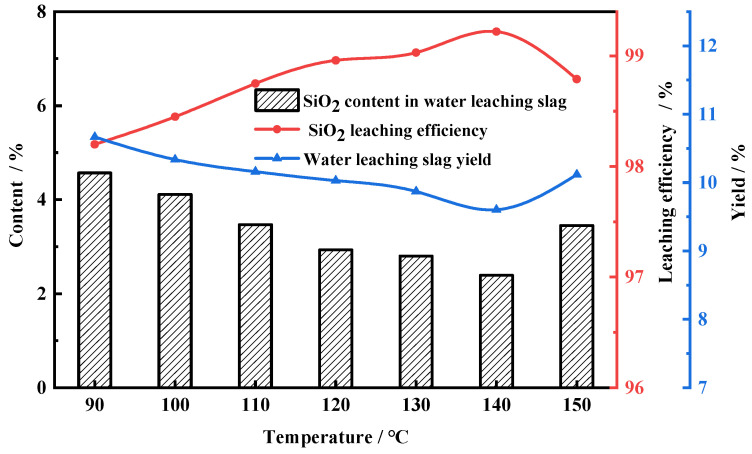
Leaching efficiency of FNS-leaching residue at different roasting temperatures. Roasting conditions: NH_4_F mass ratio, 4.3:1; roasting time, 2 h. Water-leaching conditions: leaching time, 1.5 h; liquid–solid ratio, 10: 1; stirring speed, 300 r/min.

Figure 5 shows the relationship between the roasting temperature and the leaching efficiency of SiO_2_ when the NH_4_F mass ratio was fixed at 4.3:1. Obviously, the leaching efficiency increased gradually as the roasting temperature increased from 90 to 140 °C, and the yield and SiO_2_ content of water-leaching slag simultaneously decreased. With the temperature increasing from 140 to 150 °C, the water-leaching efficiency showed a decreasing trend. This might be due to the reaction between NH_4_F and ferric oxide forced by the excessive temperature, which is adverse for the fluoridation reaction during the roasting process.

**Figure 6:**
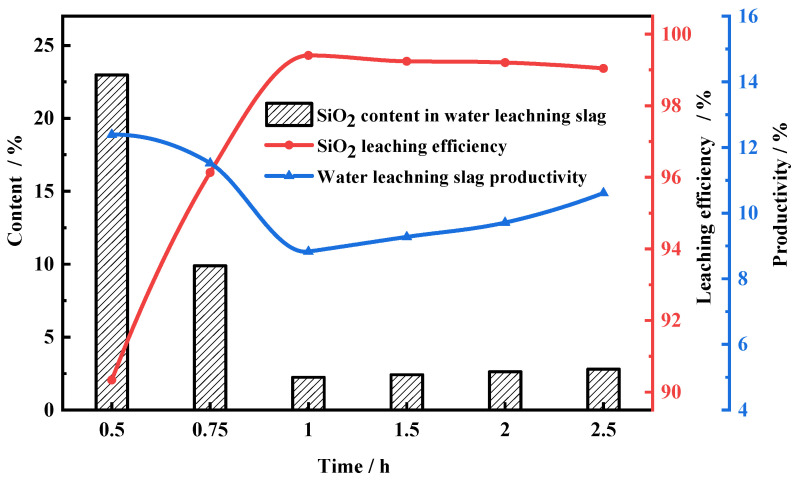
Leaching efficiency of FNS-leaching residue for different roasting time. Roasting conditions: roasting temperature 140 °C; NH_4_F mass ratio, 4.3:1. Water-leaching conditions: leaching time, 1.5 h; liquid–solid ratio, 10: 1; stirring speed, 300 r/min.

Figure 6 shows the relationship between the roasting time and the leaching efficiency of SiO_2_ when the roasting temperature was fixed at 140 °C. Evidently, the leaching efficiency increased as the roasting time increased from 0.5 to 1 h; meanwhile, the yield and SiO_2_ content of leaching slag also obviously decreased. When the roasting time was more than 1 h, the water-leaching efficiency remained roughly constant. Hence, the suitable roasting conditions are to be mixed with NH_4_F (the mass ratio is 4.3:1) and roasted for 1 h at 140 °C.

**Figure 7:**
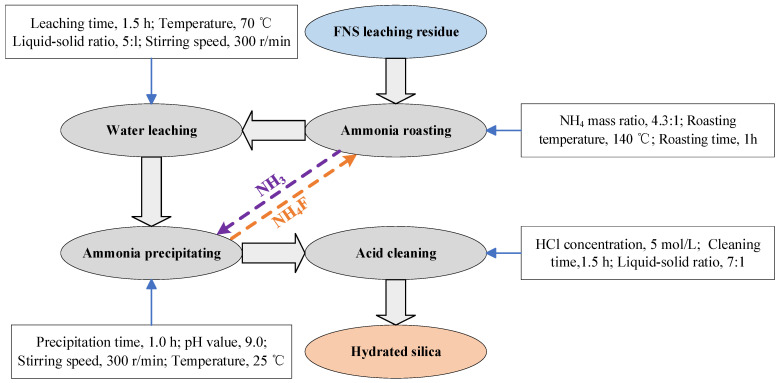
The entire process for the synthesis of hydrated silica.

On the basis of the above research, condition experiments of water-leaching, ammonia-precipitating, and acid-cleaning processes were also carried out. The hydrated silica synthesis process as well as the optimum conditions of different procedures are shown in Figure 7. The SiO_2_ recovery and content of different procedures are presented in Table 2.

It can be seen from Table 2 that the purification of hydrated silica with SiO_2_ 99.94% grade and 95.11% recovery can be obtained through the low-temperature roasting and subsequent procedures. In addition, the cyclic utilization of NH_3_ and NH_4_F for the ammonia-roasting procedure and ammonia-precipitating procedure makes the entire synthesis process more environmentally and economically friendly, which is also an advantage for the facile synthesis process when compared with other technologies.

**Figure 8:**
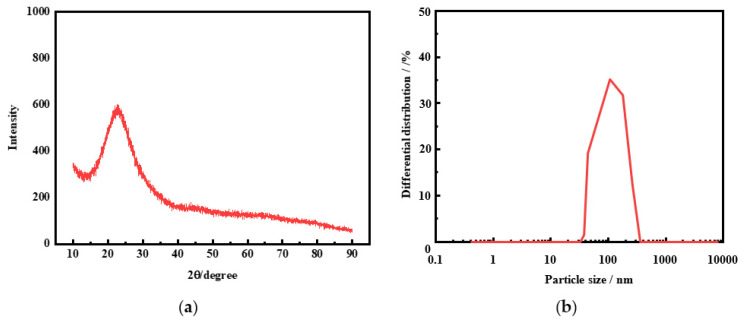
XRD analysis (**a**) and volume-weighted particle size distribution (**b**) of the purification hydrated silica.

The XRD patterns and particle size distribution of the synthetic hydrated silica are presented in Figure 8a and Figure 8b, respectively. As shown in Figure 8a, the broad diffraction peaks at the 2θ range of 20~30° are the typical characteristic peaks of the glass phase, indicating the highly purified composition of hydrated silica. It can be seen from Figure 8b that the particle size distribution of hydrated silica is relatively uniform and concentrative, and the D_50_ value is 103.66 nm, which meets the requirement of nanoparticle size for hydrated silica.

**Figure 9:**
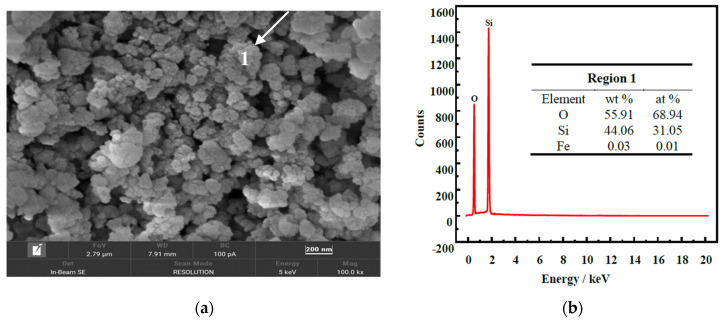
SEM images (**a**) and EDS spectrum (**b**) of the purified of hydrated silica.

Figure 9 shows the results of the SEM and EDS analyses. The morphology of hydrated silica (Figure 9a) indicates that the particle size and surface appearance is obviously different from the initial FNS-leaching residue (seen in Figure 2a), the dense structure with irregular shape was translated to spheroidal particles in micro-nanoscale. It can be seen from Figure 9b that the content of silicon and oxygen elements are more than 99.9%; meanwhile, the impurity elements such as magnesium, aluminum, calcium, etc., are almost completely removed. 

### 2.3. Thermodynamic Analysis of the Ammonium Fluoride Roasting Process

The thermodynamic analysis of the ammonium fluoride roasting process was performed by varying the temperature from 0 to 1000 °C. Apart from amorphous silica, the majority of impurities are mainly metallic oxide such as magnesium, aluminum, calcium, iron oxide, etc. According to the aforementioned mineralogical characteristics, the possible reactions are as follows [26,30,31]:

(1) $${\mathrm{SiO}}_{2}+{6\mathrm{NH}}_{4}F\;\to {\left({\mathrm{NH}}_{4}\right)}_{2}{\mathrm{SiF}}_{6}+{4\mathrm{NH}}_{3}(g)+{2H}_{2}O$$

(2) $$\mathrm{MgO}+{2\mathrm{NH}}_{4}F\;\to \;\mathrm{Mg}F_{2}+{2\mathrm{NH}}_{3}(g)+H_{2}O$$

(3) $$\;{\;\mathrm{Fe}}_{2}O_{3}+{6\mathrm{NH}}_{4}F\;\to 2{\mathrm{FeF}}_{3}+{6\mathrm{NH}}_{3}(g)+{3H}_{2}O$$

(4) $${\;\mathrm{Al}}_{2}O_{3}+{6\mathrm{NH}}_{4}F\;\to \;2{\mathrm{AlF}}_{3}+{6\mathrm{NH}}_{3}(g)+{3H}_{2}O$$

(5) $$\mathrm{CaO}+{2\mathrm{NH}}_{4}F\;\to \;\mathrm{Ca}F_{2}+{2\mathrm{NH}}_{3}(g)+H_{2}O$$

**Figure 10:**
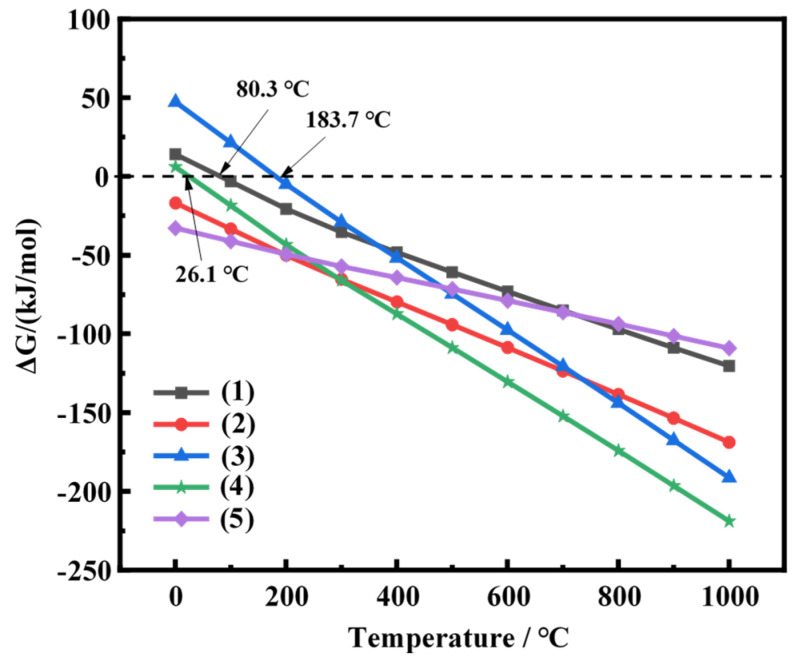
Relationship between ΔG and temperature for the reactions (calculated by HSC 6.0).

In the standard state, the Gibbs free energies ΔG of reactions (1)–(5) at different temperatures are calculated based on the fundamental thermodynamic parameters, and the results are presented in Figure 10; ΔG gradually decreased as the temperature increases. Compared to reactions (1), (3), and (4), the ΔG of reactions (2) and (5) are negative at lower temperatures, indicating that the ammonium fluoride could react with calcium oxide and magnesium oxide at ambient temperature (25 °C). When the temperature is higher than 80.3 °C, the ΔG of reaction (1) changes to the negative value, indicating that the amorphous silica can react with ammonium fluoride spontaneously at this condition. The results of thermodynamic analysis demonstrated that ammonium fluoride could effectively break the Si–O bonds and the amorphous silica were converted into water-soluble fluoride salts, realizing a low-temperature roasting process for FNS-leaching residue.

### 2.4. Mechanism of Ammonium Fluoride Roasting

**Figure 11:**
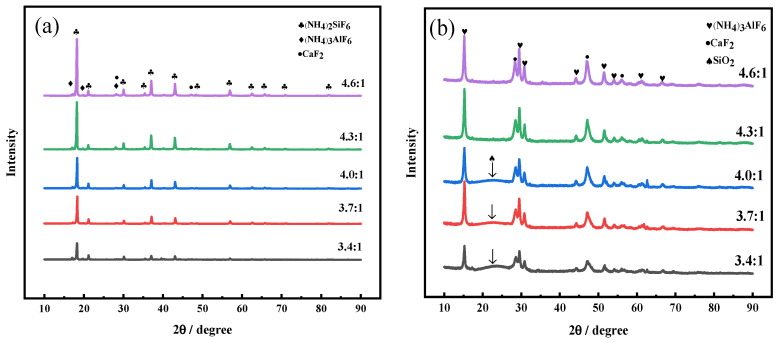
XRD analysis of ammonium fluoride roasting slag at different mass ratio of NH_4_F (**a**) before and (**b**) after water leaching. Roasting conditions: roasting temperature 140 °C, roasting time 2 h.

To investigate the fluoride reaction products of amorphous silica and ammonium fluoride during the roasting process, the crystalline characteristics of roasting products were analyzed by XRD and the results are shown in Figure 11.

It can be seen from Figure 11a that the new peaks of crystalline phase appeared in the ammonium fluoride roasting products before water leaching. When the mass ratio of NH_4_F increases from 3.4:1 to 4.3:1, the diffraction peaks of (NH_4_)_2_SiF_6_ are obviously strengthened, demonstrating that ammonium fluoride could react with the amorphous silica composition in FNS-leaching residue. Furthermore, the roasting products have essentially the same diffraction peaks with an increasing mass ratio, from 4.3:1 to 4.6:1, indicating that NH_4_F was enough for fluoride reaction at mass ratio of 4.3:1, which is in line with the results of low-temperature roasting experiments. Meanwhile, the peaks of (NH_4_)_3_AlF_6_ and CaF_2_ also can be observed in the spectrum, which might be ascribed to the residuary metal oxide substances reacting with ammonium fluoride. 

As shown in Figure 11b, the (NH_4_)_2_SiF_6_ diffraction peaks of the roasting products disappeared after water leaching, indicating that the fluoride salts (NH_4_)_2_SiF_6_ could be dissolved during the leaching process, whereas the peaks of (NH_4_)_3_AlF_6_ and CaF_2_ can still be observed after water leaching, which might be attributed to their lower solubility. In addition, the broad diffraction peaks of amorphous silica appeared when the mass ratio of ammonium fluoride was lower than 4.3:1, indicating the existence of incompletely reacted amorphous silica, which is also consistent with the results of low-temperature roasting experiments.

**Figure 12:**
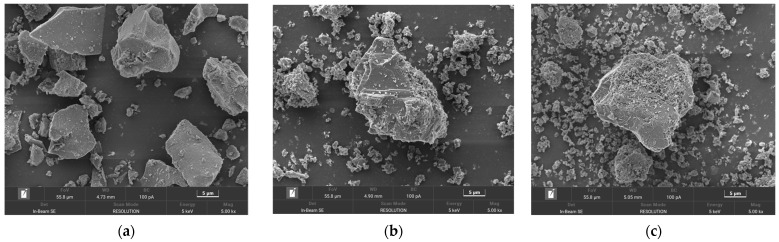

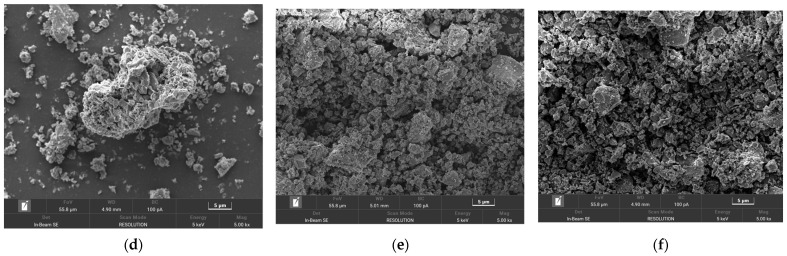
SEM images of (**a**) original FNS-leaching residue, and low-temperature roasting products at different mass ratio of NH_4_F: (**b**) 3.4:1, (**c**) 3.7:1, (**d**) 4.0:1, (**e**) 4.3:1, (**f**) 4.6:1. Roasting conditions: roasting temperature 140 °C, roasting time 2 h.

The morphology of original FNS-leaching residue and low-temperature roasting products at different mass ratios of NH_4F_F were examined by SEM and shown in Figure 12. It can be seen from Figure 12a that the FNS-leaching residue is mainly composed of particles with a smooth surface and irregular shape. However, as shown in Figure 12b, the roasting products were translated onto the corrosion surface when the mass ratio of NH4F is 3.4:1, indicating that the surface layer of amorphous silica was decomposed by ammonium fluoride. With the mass ratio of NH_4_F increasing to 4.0:1, as shown in Figure 12d, the lumpy structure of particles was damaged and the micropores formed, which can be ascribed to ammonium fluoride destroying the surface layer and decomposing the inner structure further. When the mass ratio of NH_4_F was more than 4.3:1, the lumpy structure was entirely destroyed and changed to the scraps which were stuck together. Based on the aforementioned results, the roasting products are mainly translated from amorphous silica to (NH_4_)_2_SiF_6_ at this condition.

**Figure 13:**
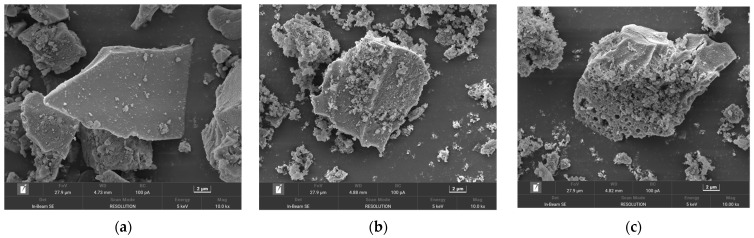

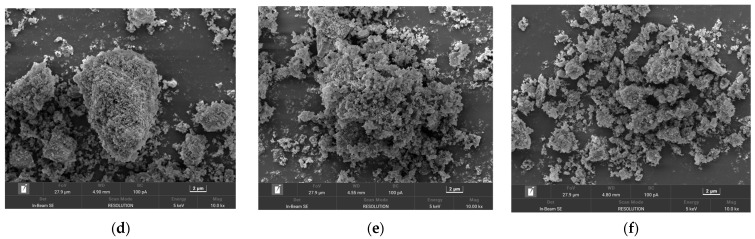
SEM images of (**a**) original FNS-leaching residue, and low-temperature roasting products after water-leaching treatment at a different mass ratio of NH_4_F: (**b**) 3.4:1, (**c**) 3.7:1, (**d**) 4.0:1, (**e**) 4.3:1, (**f**) 4.6:1. Roasting conditions: roasting temperature 140 °C, roasting time 2 h.

The morphologies of the roasting products after water-leaching treatment are shown in Figure 13. The mass ratio of ammonium fluoride can clearly influence the microstructure of water-leaching slag. When the mass ratio of NH_4_F was 3.4:1, the morphology of water-leaching slag was similar with that of original FNS-leaching residue (seen in Figure 13a,b), except for the formation of some microcracks and micropores. With the increase in the NH_4_F mass ratio, the abundant formation of cracks and pores can easily be observed from the water-leaching slag (seen in Figure 13c,d). This phenomenon is mainly due to the ammonium fluoride further reacting with the internal structure of amorphous silica and the formed (NH_4_)_2_SiF_6_ dissolving during the water-leaching process. When the mass ratio of NH_4_F was more than 4.3:1, through the treatment of low-temperature roasting and water leaching, the basic structure of amorphous silica was fully destroyed and decomposed to few disintegrating slags, which is consistent with the results of XRD analyses and water-leaching experiments.

**Figure 14:**
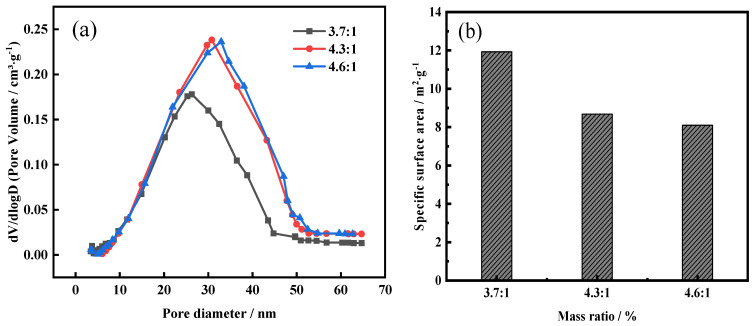
The pore size distribution (**a**) and specific surface area (**b**) of low-temperature roasting products after water-leaching treatment at different mass ratios of NH_4_F. Roasting conditions: roasting temperature 140 °C, roasting time 2 h.

To further investigate the micro-structure of water-leaching slag, the pore size distribution and specific surface area of slag were examined and the results are shown in Figure 14. It can be seen that, with the increase in the NH_4_F mass ratio, the pore size gradually increased, whereas the specific surface area presented an obvious downward trend. When the mass ratio of NH_4_F was 3.7:1, the amorphous silica in FNS-leaching residue was partly decomposed and dissolved, and the forming pore size of water-leaching slag concentrated on a range of 20–30 nm. With the NH_4_F mass ratio increasing to greater than 4.3, the residual amorphous silica was decomposed and dissolved completely, and the pore size further expanded to a range of 25–45 nm. The phenomenon that multiple micropores translated to minority macropores also supports the specific surface area decreasing with the increase in the NH_4_F mass ratio. It can be conducted that the ammonium fluoride can obviously strengthen the low-temperature roasting and water-leaching process.

**Figure 15:**
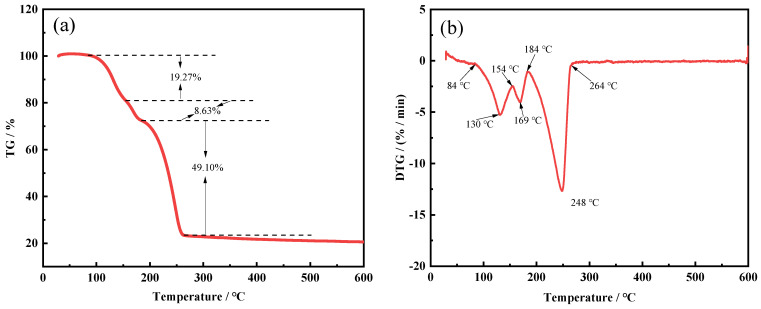
TG curve (**a**) and DTG curve (**b**) of the mixture of FNS-leaching residue and NH_4_F at a mass ratio of 4.3:1.

The thermochemical behavior of ammonium fluoride mixed with the FNS-leaching residue at a mass ratio of 4.3:1 was shown in Figure 15, and can be divided into three significant weight losses. The first significant weight loss occurred in the range from 85 to 155 °C, and that was approximately 19.27%. According to the results of the thermodynamic analysis (seen in Figure 11), the ammonium fluoride could react with the amorphous silica, calcium oxide, and magnesium oxide in the range of 85 to 155 °C, which is also supported by the weight loss on this condition. The secondary weight loss occurred in the temperatures range from 155 to 184 °C, and the weight loss was only about 8.63%. This might be attributed to the reaction of ammonium fluoride with iron oxide, which required a higher temperature to realize the spontaneous reaction. The third significant weight loss occurred in the temperature ranging from 184 to 274 °C and was nearly 49.10%. In consideration of the decomposition temperature of (NH_4_)_2_SiF_6_, the larger and faster weight loss might be due to the formed (NH_4_)_2_SiF_6_ further decomposed to a gaseous substance over an excessive temperature. This is also consistent with the rapid increase in weight loss, which reached a maximum at approximately 284 °C.

## 3. Materials and Methods

### 3.1. Materials

The raw materials’ leaching residue is the residue of FNS after extracting Mg, Fe and other valuable metals. The FNS was provided by a ferronickel metal smelting plant in Hebei Province, China. The ammonium fluoride, ammonium hydroxide, and hydrochloric acid were all analytical grade and provided by Sinopharm Group Chemical reagent Co., LTD, Shanghai, China; they were used as activator, precipitator, and acid cleaner for the synthesis process of hydrated silica, respectively. In addition, all solutions were produced or diluted using deionized water.

### 3.2. Experimental Process

**Figure 16:**
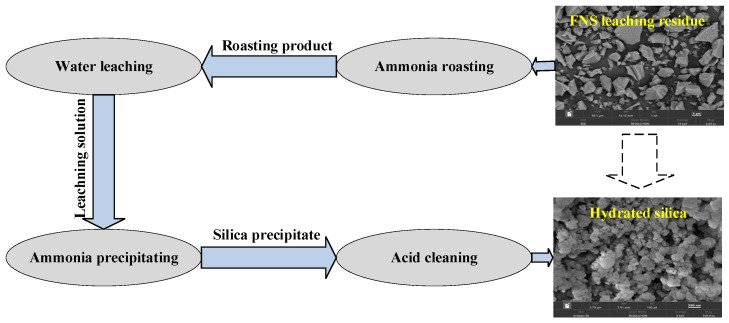
The schematic of experimental process for the synthesis of hydrated silica derived from ferronickel slag-leaching residue.

A diagram schematic of experimental process is shown in Figure 16. The critical step for the entire process is ammonium fluoride roasting. The first step was to convert the amorphous silica components into water-soluble products by low-temperature roasting; the second step was to dissolve the water-soluble products by water leaching; the third and fourth step involved precipitating the silica components in the solution and improving the hydrated silica quality through acid cleaning, respectively. 

Ammonium fluoride roasting was performed in a muffle furnace. A mixture of ammonium fluoride and FNS leach residue at various mass ratios was placed in alumina crucibles and heated to certain temperature at a velocity of 10 °C/min. After roasting for a certain time, the roasting solid samples, which were used for water leaching, were cooled down to ambient temperature (25 °C); meanwhile, the flue gasses were disposed of by water adsorption.

Then, the roasting products were leached using distilled water at a certain temperature with an applicable liquid–solid ratio. The leaching slurry was filtered and washed repeatedly to obtain the leaching solution and residue, and the leaching solution was used as the raw solution for subsequent ammoniating precipitation. The leaching efficiency *α* can be calculated by Equation (6).

(6) $$\alpha =\frac{m_{1}\times x_{1}-m_{2}\times x_{2}}{m_{1}\times x_{1}}\times 100\%$$

where *α* is the leaching efficiency of SiO_2_; *m*_1_ and *m*_2_ are the weight of roasting solid samples before and after water leaching (g), respectively; and *x*_1_ and *x*_2_ are the SiO_2_ content of roasting solid samples before and after water leaching (wt%), respectively.

Finally, the leaching solution containing rich silica is treated with ammonium hydroxide at appropriate conditions to realize the silica precipitation, and the crude hydrated silica can be obtained by the treating of artificial aging and filtration. To further improve the purity of crude hydrated silica products, a certain concentration of hydrochloric acid was used to further dissolve the impurities to obtain the purified hydrated silica products.

### 3.3. Characterization Methods

The particle size distribution of the samples was determined by a Malvern Mastersizer 2000 laser particle size analyzer. The pore properties and specific surface area of ammonium fluoride-roasting products were recorded on nitrogen adsorption–desorption isotherm measurements at 77 K (Micromeritics Tristar II 3020, GA, Norcross, U.S.). The chemical composition of solid samples was examined by an X-ray fluorescence spectrometer (Shimadzu XRF-1800, Kyoto, Japan), with an Rh target and a maximum power of 60 kV, 140 mA. An X-ray fluorescence spectrometer (Rigaku Ultima IV, Tokyo, Japan) was used to analyze the physical phase. The test conditions: tube voltage 40 kV, tube current 40 mA, Cu target Kα rays (λ = 15.406 nm), step size 0.026°, scanning speed 20°/min, scanning range 2θ = 10–90°. Morphological analyses were conducted using scanning electron microscopy (Carl Zeiss Gemini 300, Jena, Germany). Meanwhile, elemental composition was analyzed with an Oxford Xplore 30 energy spectrum analyzer. The detector was an SE2 secondary electron detector and the acceleration voltage for the morphology and energy spectrum was 3 kV and 15 kV, respectively. A Mettler TGA/DSC3+ simultaneous thermal analyzer (Zurich, Switzerland) was used to analyze the TG-DSC curves of the samples, where the test temperature ranges from room temperature to 600 °C and the temperature increase rate is 10 °C/min in an air atmosphere.

## 4. Conclusions

In this study, a novel process, including ammonium fluoride (NH_4_F) roasting, water-leaching, ammonia precipitating, and an acid cleaning procedure, was proposed for the synthesis of hydrated silica derived from ferronickel slag (FNS)-leaching residue. The optimum conditions are: NH_4_ mass ratio of 4.3:1; roasting temperature of 140 °C during roasting procedure; leaching time of 1.5 h; leaching temperature of 70 °C during the water-leaching procedure; precipitating time of 1.0 h; solution pH value of 9.0 during the ammonia precipitating procedure; cleaning time of 1.5 h; and HCl concentration of 5 mol/L during the acid cleaning procedure. The characterization results of purified hydrated silica confirmed that its grade and recovery reached 99.68% and 95.11%, respectively.

The thermodynamic, X-ray diffraction, scanning electron microscopy, and thermogravimetry analyses were used to investigate the activation mechanism of NH_4_F for low-temperature roasting. The results demonstrated that the Si–O bonds in amorphous silica were effectively broken through ammonium fluoride activation and the amorphous silica was converted to water-soluble fluoride salts ((NH_4_)_2_SiF_6_) during the roasting process, in which the water leaching can realize the impurities removing and silicon enrichment at the same time. This work provides a meaningful reference for further studies on the facile synthesis of hydrated silica with similar mineral compositions.

## Figures and Tables

**Table 1:** Major composition in the leaching residue of FNS analyzed by XRF.

Chemical Composition	SiO_2_	CaO	Fe_2_O_3_	Al_2_O_3_	MgO	Cr_2_O_3_	SO_3_	MnO
Contents (wt%)	81.34	6.84	4.21	3.98	1.59	0.73	0.31	0.52
Chemical Composition	TiO_2_	K_2_O	Na_2_O	NiO	SrO	Cl	ZnO	P_2_O_5_
Contents (wt%)	0.2	0.11	0.09	0.02	0.02	0.02	0.01	0.01

**Table 2:** The SiO_2_ content and recovery of different products in the entire process.

Products	SiO_2_ Content		SiO_2_ Recovery/%	
	XRF/wt%	ICP/g·L^−1^	Separate	Cumulative
FNS-leaching residue	81.34	79.62	100	100
Roasting products	29.47	28.00	100	100
Water-leaching solution	--	28.91	99.53	99.53
Crude hydrated silica	94.49	92.35	96.12	95.67
Purified hydrated silica	99.94	99.68	99.41	95.11

## Data Availability

Data are contained within the article.
